# The Impact of Toxic Leadership on Nurse Retention: A Scoping Review

**DOI:** 10.3390/healthcare13182341

**Published:** 2025-09-17

**Authors:** Eleni Tsapnidou, Maria Moudatsou, George Katharakis, Sofia Koukouli, Michael Rovithis, Martha Kelesi, Areti Stavropoulou

**Affiliations:** 1 Department of Nursing, School of Health, Faculty of Health and Care Sciences, University of West Attica, 12243 Egaleo, Greece; gkatharakis@uniwa.gr (G.K.); mkel@uniwa.gr (M.K.); astavropoulou@uniwa.gr (A.S.); 2 Department of Social Work, Faculty of Health Sciences, Hellenic Mediterranean University, 71410 Heraklion, Greece; moudatsoum@hmu.gr (M.M.); koukouli@hmu.gr (S.K.); 3 Department of Business Administration and Tourism, School of Management and Economics Sciences, Hellenic Mediterranean University, 71410 Heraklion, Greece; rovithis@hmu.gr

**Keywords:** toxic leadership, nursing staff, staff retention, job satisfaction

## Abstract

**Background/Objectives**: Toxic leadership has arisen as a matter of serious concern within the nursing profession, with growing evidence linking it to diminished job satisfaction, ineffective conflict management, and weakened organizational commitment. These effects not only compromise nurse retention but also threaten the quality of patient care and overall healthcare outcomes. This scoping review aimed to examine the impact of toxic nursing leadership on staff retention by synthesizing evidence from existing literature and a broad range of published studies. **Methods**: A comprehensive literature search was conducted across multiple databases, including PubMed/MEDLINE, Scopus, CINAHL and Science Direct databases yielding 1356 articles. Of these, 18 met the predefined inclusion criteria. The scoping review followed the six-stage methodological framework proposed by Arksey and O’Malley. Thematic analysis identified two core categories: (a) key dimensions shaping perceptions of toxic leadership and (b) the impact of toxic leadership on nursing staff retention. **Results**: The findings reveal that toxic leadership contributes to organizational silence, emotional exhaustion, diminished psychological safety, and low professional commitment. Such behaviors not only jeopardize nurse engagement and productivity but also negatively affect patient safety and care quality. In contrast, leadership styles such as transformational and transactional leadership are associated with higher job satisfaction, reduced burnout, and improved retention outcomes. **Conclusions**: This review underscores the need for healthcare organizations to identify and address toxic leadership behaviors promptly. By promoting supportive and ethical leadership styles, institutions can foster a healthier workplace, improve nurse retention, and ultimately enhance the quality of care. The study offers practical implications for healthcare administrators, emphasizing leadership development.

## 1. Introduction

Leadership is a complex and evolving concept with various definitions. It is often defined as an influencing process aimed at achieving goals, where leaders mobilize others to strive for shared aspirations [1]. It is also described as the ability to establish and accomplish objectives, react effectively to challenges and empower others within an organization [2]. The ambiguity and multiplicity of leadership pose significant challenges. The concept is often conflated with management, although some argue that leadership and management are distinct, yet complementary roles [1].

Nursing leadership is often described as the process of influencing and guiding nurses to attain joint outcomes, with a focus on improving patient outcomes and enhancing the work environment [3,4]. Key attributes of nursing leadership include the ability to motivate, support and develop team members, as well as to foster decision-making [5]. Effective nursing leadership is linked to better patient outcomes, reduced medical errors and enhanced patient safety. It encourages evidence-based practices, increases staff engagement, and promotes collaboration with other healthcare professionals [6,7,8]. Positive leadership styles such as relational leadership promote the nurses’ well-being and productivity, while addressing challenges and empowering nurses to strive for excellence and quality care [6,9].

On the other hand, negative leadership behaviors, such as toxic leadership, are linked to decreased job satisfaction due to the stress caused by exploitative managerial actions. These may lead to higher turnover rates and financial losses for the healthcare organizations [10,11]. Toxic leadership affects team dynamics, leading to poor conflict management and reduced organizational commitment [12]. It may also result in a negative working environment, poor quality of care and increased frequencies of adverse clinical events. Understanding abusive leadership helps in developing strategies to mitigate its effects, as this leadership style may undermine workforce stability, productivity, staff resilience, and patient safety [13]. This scoping review synthesizes evidence from multiple countries, addressing a critical gap by offering a broader perspective on toxic leadership in healthcare, rather than limiting the analysis to a single organization or setting. Periodically consolidating this literature is essential, as the global nursing shortage increasingly threatens quality of care, patient safety, and staff well-being. This study focuses on how healthcare professionals identify key dimensions and behaviors of toxic leadership and how toxic leadership influences nurse retention in healthcare organizations.

### 1.1. Toxic Leadership and the Toxic Leader

Toxic leadership is a detrimental style of leadership characterized by harmful behaviors and attitudes that negatively impact organizations and individuals. Smidt [14] suggested that toxic leadership encompasses five core elements: abusive supervision, authoritarianism, narcissism, self-promotion, and behavioral inconsistency.

The concept of toxic leadership was introduced in 1996, initially explored within corporate and military settings. It was not until 2007 that empirical research began to connect abusive leadership with higher education systems, highlighting its influence on leadership culture in these environments [15,16]. Over the years, the research has expanded to include various organizational contexts, including education and manufacturing [17].

Dysfunctional leadership is marked by behaviors such as abuse, bullying, and destructive actions that create a harmful work environment. These leaders often aim to conceal their incompetence and maintain control, leading to increased ambiguity and confusion within the organization [18,19]. They often exhibit behaviors of being autocratic, manipulative, controlling, deceitful, and callous. These demeanors distinguish them from merely difficult leaders and contribute to a toxic work environment [18,20]. The personality traits associated with toxic leaders include Machiavellianism, narcissism, and psychopathy, which contribute to organizational failures [21]. These dispositions often lead to unethical decision-making and a focus on personal gain over organizational well-being [21,22].

Destructive leadership negatively affects job satisfaction, commitment, and turnover intention, with organizational culture playing a mediating role in these outcomes [17]. It undermines employee well-being, health, morale, and productivity, ultimately impacting organizational effectiveness [23]. Oppressive leadership in higher education exerts harmful effects on faculty, staff, and students. Such practices lead to emotional strain, lower job satisfaction, and widespread organizational dysfunction. [22]. Toxic leadership, as a destructive model, is characterized by behaviors—including manipulation, pressure, and recognized humiliation—that decompose organizational culture, undermine employee morale, and compromise institutional efficacy.

### 1.2. Nursing Staff Retention

The shortage of nurses is a critical issue affecting healthcare systems globally. This scarcity has been persistent and is exacerbated by various factors. The aging of nursing staff leads to a gap in the workforce as there are not enough new entrants to fill these positions. Declining enrolment in nursing programs further exacerbates this issue [24]. The strain of continuous high stress working conditions leads to professional burnout, ultimately increasing nurse turnover and diminishing workforce sustainability [25,26]. Economic challenges and other social factors, such as the impact of the COVID-19 pandemic, have intensified the shortage by increasing job vacancies and affecting the well-being of the existing nurses [27,28].

Nursing staff are integral to maintaining a safe hospital environment and ensuring high-quality patient care. Their engagement in hospital safety strategies is crucial for achieving optimal patient safety outcomes [29]. Adequate nursing staff levels are associated with better patient safety outcomes, such as reduced fall rates and hospital-acquired pressure ulcers. Retaining experienced nurses also plays a significant role in patient safety [30]. Instead, low nurse staffing levels are consistently associated with adverse patient outcomes. These include higher rates of in-hospital mortality, hospital-acquired infections, medication errors, and patient falls [31,32,33,34]. Studies have shown that inadequate staffing leads to increased missed care, which directly impacts the quality of care and patient safety [35,36,37]. Additionally, personnel shortages in emergency departments have been linked to longer door-to-discharge times and an increased number of patients leaving without being seen [38].

Nursing staff retention is a critical issue in healthcare, influenced by a variety of factors. A positive work environment and high job satisfaction are crucial for retaining nursing staff. Factors such as supportive management and leadership, group cohesion, and reduced job stress contribute significantly to retention [39,40,41,42]. Factors such as staffing levels, career advancement opportunities, and financial remuneration contribute significantly to the decrease in the turnover levels [43,44]. Nurses who feel appreciated and supported by their managers and peers are more likely to stay. Recognition of their work and contributions is vital for retention [39,44].

## 2. Materials and Methods

### 2.1. Aim of the Study

The aim of this scoping review was to examine the impact of toxic leadership on nurses’ retention. This research specifically aims to respond to the following research questions that were developed following the PIO (Population, Intervention, Outcome) framework where Population is the Nurses, Intervention is the toxic leadership and Outcome is the nursing staff retention:

Q1: What are the key dimensions and behaviors associated with toxic leadership in nursing as identified by the healthcare professionals?

Q2: How does toxic leadership influence nursing staff on their career decisions and retention in healthcare organizations?

### 2.2. Design

A scoping review is a rigorous exploratory methodology designed to systematically map the range of existing literature on a defined research topic. Unlike traditional systematic reviews, which focus on answering highly specific questions, scoping reviews employ a structured yet flexible approach to identify key concepts, evidence gaps, and emerging trends within a field. By applying predefined criteria, this methodology facilitates a comprehensive analysis and synthesis of existing literature, enabling researchers to address their study’s broader research questions [45]. In alignment with well-known best practices, this study adopts the systematic six-stage framework originally developed by Arksey and O’Malley [45], ensuring methodological transparency and accuracy throughout the review process.

### 2.3. Identifying Relevant Studies

*Eligibility criteria.* This analysis incorporated peer-reviewed studies examining toxic leadership in healthcare organizations, including quantitative (cross-sectional, longitudinal), qualitative, and mixed-methods research designs. The inclusion criteria required that studies (a) specifically analyze toxic leadership behaviors and their organizational impacts, (b) be published in English between 2019 and 2025 to ensure contemporary relevance, and (c) provide empirical evidence from healthcare contexts. Studies that (a) addressed leadership generically without focusing on toxic behaviors, (b) were published prior to 2019, except those that were cited in the conceptual framework, (b) lacked peer-review validation, or (c) were unavailable as full-text articles, were excluded. Specific consideration was given to excluding studies that conflated toxic leadership with other negative leadership styles (e.g., laissez-faire or incompetent leadership) to maintain conceptual clarity. A thorough selection process ensured inclusion of the most relevant and methodologically robust research on the harmful impacts of toxic leadership in healthcare organizations.

*Information sources and search strategy*. A comprehensive, multi-database search strategy was executed across PubMed/MEDLINE, Scopus, CINAHL, and Science Direct to capture the full spectrum of research on toxic leadership in healthcare backgrounds. The search incorporated an in-depth set of leadership-related terms including “toxic leadership”, “abusive leadership”, “negative leadership”, “oppressive leadership”, and “destructive leadership,” combined with healthcare-specific terms through Boolean operators (AND/OR/NOT) to ensure methodological consistency. The search strategy specifically targeted studies examining these leadership styles’ impacts on nursing staff and healthcare organizations, with all search parameters and term combinations systematically documented in Table 1 to ensure transparency and reproducibility.

### 2.4. Study Selection

**Figure 1:**
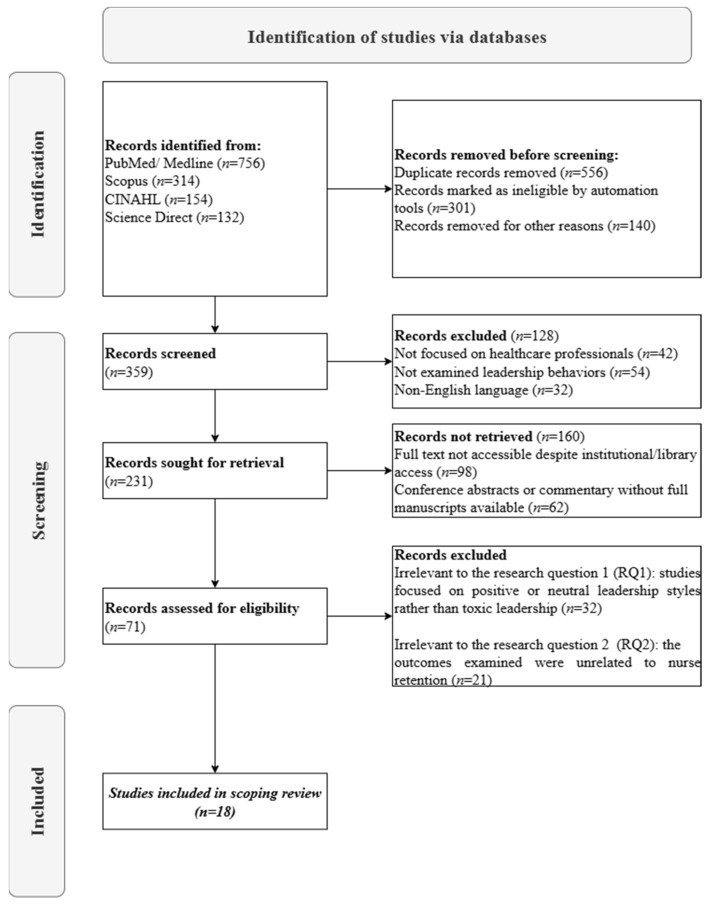
PRISMA Flow Diagram of Study Selection.

The study selection process followed a thorough, systematic approach, as detailed in the PRISMA flowchart (Figure 1) [4]. Initial database searches across PubMed/MEDLINE, Scopus, CINAHL, and Science Direct yielded 1356 records. After removing duplicates (*n* = 556) and records deemed ineligible through automated tools and manual screening (*n* = 441), 359 studies underwent full-text assessment. Of these studies, 231 were sought for retrieval, with 160 unavailable, leaving 71 studies for eligibility evaluation. Following a stringent review, 53 studies were excluded—32 for irrelevance to Research Question 1 (RQ1) and 21 to Research Question 2 (RQ2)—resulting in 18 studies that met all inclusion criteria. These studies were selected for their explicit focus on toxic leadership and its detrimental impacts within healthcare backgrounds. This deliberate selection ensures the review captures the most relevant evidence on toxic leadership’s consequences, providing a necessary contrast to dominant narratives of positive leadership.

### 2.5. Charting the Data

In accordance with Arksey and O’Malley’s established framework [45], a carefully designed data extraction and synthesis process was implemented to systematically examine toxic leadership in healthcare contexts. The methodology began with the development of a structured data extraction table designed to capture critical elements from each study, including specific toxic leadership behaviors, their healthcare-specific consequences, and key methodological characteristics. To ensure reliability, a pilot test on a subset of four studies (approximately 22% of the total studies) was conducted. During this pilot, two researchers independently extracted data using the preliminary table. The process revealed minor ambiguities in how certain toxic leadership behaviors and outcomes were classified. Based on these findings, the extraction criteria were refined. Inter-rater reliability (IRR) was then calculated by comparing the two researchers independent coding decisions across all extraction fields. Cohen’s kappa coefficient was used, which adjusts for chance agreement, and the resulting score exceeded 0.85, indicating strong agreement. Following this refinement, the finalized extraction framework was applied consistently across all 18 studies. Three researchers with expertise in organizational behavior and healthcare leadership independently extracted data from the allocated studies. Due to a high initial inter-researcher agreement, few significant discrepancies arose. The limited discrepancies that did occur were resolved through systematic consensus discussions, and all decisions were documented to maintain transparency throughout the analysis process. The final synthesized data, presented in Appendix A, provides a comprehensive overview of the included studies. This includes detailed information on authorship, publication dates, geographic focus, research objectives, methodological approaches, and key findings related to toxic leadership’s impact on healthcare environments (see Table A1). This methodical approach aligns with best practices in scoping reviews and the consensus-based validation process ensures the findings are both reproducible and accurate.

### 2.6. Data Analysis

The extracted data were systematically analyzed within two conceptual categories: (a) the key dimensions shaping perceptions of toxic leadership in healthcare environments, and (b) its measurable impact on nursing staff retention. These thematic dimensions were chosen, in order to reveal critical patterns across the literature, with the first thematic category exposing how nurses identify and experience toxic leadership through specific behavioral manifestations (e.g., psychological manipulation, bullying, and inconsistent decision-making), while the second category documented its devastating consequences on workforce stability (including increased turnover intentions, emotional exhaustion, and premature career abandonment). Three lead researchers conducted the primary analysis coding and categorizing findings, followed by iterative consensus-building sessions with the entire research team to verify emerging themes. This collaborative methodology supported robust interpretation of the findings while preserving the contextual integrity of the original studies. The final synthesis was structured according to PRISMA-ScR guidelines [46]. This scoping review was registered on the Open Science Framework platform on 25 July 2025 (Registration DOI https://doi.org/10.17605/OSF.IO/UWY4G).

### 2.7. Consultation

Preliminary findings were presented to critical evaluation through discussions with the research team to ensure interpretive validity. Following this, a structured validation process with four external stakeholders from the healthcare sector was conducted. These individuals were selected based on their extensive leadership experience and expertise in organizational management within healthcare environments. They were then invited to critically assess the clarity, accuracy, and significance of these findings, offering feedback on whether the results resonated with their professional experiences. Additionally, they highlighted practical considerations for addressing toxic leadership in institutional settings, such as policy adjustments, leadership training programs, and support mechanisms for affected staff. Their insights provided valuable external perspectives on both the interpretation of the results including the identified dimensions of toxic leadership and its documented effects on nursing staff retention.

## 3. Results

### 3.1. Characteristics of the Included Studies

As presented in Table 2, the included studies varied by region, publication period, methodological approach, and thematic emphasis on toxic leadership dimensions or nurse retention outcomes.

The scoping review incorporated 18 studies examining toxic leadership in healthcare, with distinct geographical and methodological patterns. Geographically, nearly half originated from Asia (44.4%, *n* = 8), followed closely by Africa (38.9%, *n* = 7), while European and North American studies were underrepresented (11.1% and 5.6%, respectively). Temporally, most publications emerged in the latter half of the review period (61.1%, *n* = 11; 2022–2025), suggesting growing research interest. Methodologically, cross-sectional designs dominated (55.6%, *n* = 10), with mixed methods approaches representing a third of studies (33.3%, *n* = 6). Thematic analysis revealed a strong emphasis on toxic leadership’s impact on nurse retention (72.2%, *n* = 13), outweighing investigations of its subjective experiences (27.8%, *n* = 5). This distribution highlights a critical research gap in understanding how toxic leadership is identified versus its measurable workforce consequences.

### 3.2. Perceptions of Toxic Leadership Key Dimensions

Concerning the views of Chinese nurses on the nature, features, and response mechanisms related to toxic managerial behaviors in nursing leadership roles, Guo et al. [47] carried out a qualitative study conducting semi-structured in-depth interviews with 12 registered nurses in China. This study pointed out that the toxic leader depicts self-centeredness, low emotional intelligence, narrow-mindedness and competitiveness. Although the nurses notice their managers’ abusive leadership traits, they choose either to tolerate it or to ignore it, as they regard it as common or inevitable. They tendentially make positive evaluations about their oppressive managers for fear of receiving punishment or vindictive behaviors, purposeful unfairness, excessive pressure and workloads. The nurses develop silence as coping mechanism, they may choose the night shift to avoid interaction with their leader, and they even self-reflect when they receive negative behaviors.

Staff withdrawal owing to corrosive leadership behaviors was also studied by Durrah et al. [48], obtaining information from 413 healthcare workers in France. This study examined the two dimensions of staff turnover, the psychological and the physical, individually and revealed that various negative leadership behaviors trigger different workforce reactions. More specifically this study concluded that authoritarian leadership is linked to physical resignation, whereas self-promotion is more influential on psychological withdrawal behaviors. Other destructive leadership characteristics were the unpredictability and uncertainty of the supervisor, which led to lower workforce engagement and eventually turnover.

Bakkal et al. [49] investigated the impact of harmful leadership on nurses’ and hospital employees’ job satisfaction and turnover intention and the mediating effect of their perceptions of dysfunctional leadership behaviors. The study was conducted in Turkey and included a sample of 658 participants. The findings of this study indicate an inverse relationship between job satisfaction and the components of toxic leadership, specifically unappreciativeness, selfishness, and self-serving behavior. The study suggests that when employees’ perception of self-respect is attacked, their self-confidence and individual performance deteriorate. Consequently, as job satisfaction declines, turnover intention tends to increase.

Ofei et al. [50] sought to evaluate the characteristics and the relationship between toxic leadership traits in nurse management and nurses’ perceived job fulfillment and productivity in the healthcare sector. Utilizing a sample of 943 nurses from various hospitals in Ghana, this study underlined intemperance, humiliation, narcissism, and self-promotion as the most common attributes of despotic leadership. The outcomes of this study also displayed a significant positive correlation between autocratic leadership behaviors and turnover intentions. Moreover, job satisfaction appears to act as a mediating factor in this relationship, suggesting that the detrimental effects of toxic leadership on retention are, in part, driven by its impact on nurses’ satisfaction at work.

In another study in Egypt [51], in which 250 nurses took part, two hospitals, a university hospital and a health insurance hospital, were compared. In this study each hospital was studied in terms of how nurses responded to adverse leadership behaviors. Results highlighted that nurses’ intent to stay was negatively affected by authoritarian and unpredictable leadership in the university hospital and by self-promoting leadership in the health insurance hospital.

### 3.3. Toxic Leadership and Its Impact on Staff Retention

Labrague L. et al. [52] in their study in 2020 in Philippines with 770 participants compared the impact of the toxic and transformational leadership on the nurses’ work attitudes. According to their findings, toxic leadership results in diminished job satisfaction, elevated stress levels, increased rates of absenteeism, and a heightened intent to leave the profession. On the other hand, transformational leadership -where managers inspire and empower their staff- leads to higher job satisfaction and lower turnover intentions.

Ofei et al. [53] in Ghana performed an investigation about the nature and effect of destructive nurse leadership conduct for nursing staff’s perceptions of job satisfaction and productivity. In this descriptive study 943 nurses took part and revealed that emotionally damaging leadership behaviors of nurse managers related to decreased psychological empowerment, job dissatisfaction, poor work performance and greater likelihood of departure from the nursing profession and the affiliated institution.

Ramdan and Eid [54] in their research in Egypt, with a sample size of 544 participants, compared the effects of the oppressive leadership styles of nurse managers on nursing staff in two ICUs. They assessed their nurse manager’s harmful leadership related to their selected conflict management methods and organizational commitment levels. The study demonstrated a positive relationship between toxic leadership and confrontational conflict management styles and a negative correlation with the collaborative dispute resolution methods. The results also indicated a negative correlation between malicious leadership and the nurses’ organizational commitment in both hospitals highlighting the significance of the leader’s behavior regarding staff retainment and sustainability.

In China, Siyal et al. [55] conducted research with 430 healthcare professionals examining the impact of abusive supervision on the employees’ performance. The results of this study underscore that destructive leadership has a negative impact on employee performance, with job satisfaction and extrinsic motivation mediating this relationship. Consequently the healthcare organization’s image was also affected by the reduction in the quality of its services, due to the lack of interest of the employees receiving negative behaviors. On the other hand, employees who were motivated and satisfied with their leadership, depicted higher quality performance.

Regarding the impact of destructive leadership on the employee health, Trépanier et al. [56] undertook research among 399 Canadian nurses. This study analyzed the mental and motivational processes involved in how tyrannical and laissez-faire leadership styles influence employee burnout, affective commitment, and job performance. According to the outcomes of this study, tyrannical leadership style is related to emotional dysregulation, low affective commitment and poor performance, which jeopardize both patient safety and organizational success. Also, laissez-faire leadership style is highly associated with autonomy frustration, burnout and low work engagement. Both negative managerial styles were found to erode motivation, self-determination, feelings of competence, self-worth and appreciation, leading ultimately to resignation figuratively or literally.

The impact of different leadership styles on healthcare personnel’s well-being was investigated by Erschens et al. [57] in Germany, using a sample of 1137 participants from all three occupational groups (physicians, nursing staff and administrative employees). The findings of this study underlined the positive effects of effective leadership as well as the negative repercussions of dysfunctional leadership. As far as the group of nurses is concerned, those who experienced transformational and transactional leadership styles, exhibited higher well-being scores than the ones who incurred destructive and laissez-faire leadership.

Low et al. [58] led a research initiative in Malaysia, in which 377 nurses took part, and pointed out the effects of abusive supervision on the nurses’ negative and absurd behaviors. More specifically this study showed that nurses who perceived injustice or discrimination had low power distance orientation and external locus of control, and were more likely to acquire counterproductive behaviors. This tendency arises from their difficulty in accepting inequalities and their attempt to restore a sense of justice. The outcomes of this study advocate, that applying unethical nursing leadership results in antisocial behaviors, reduces compliance with the rules and the nurses’ interest about organizational goals, increases organization-oriented aggression and bullying incidents.

Shipl et al. [59] studied the effect of manipulative leadership on the nurses’ followership effectiveness. In their study in Egypt participated 343 nurses and revealed, that workforce responsiveness negatively correlated with leadership practices that undermine team cohesion, due to the instability, stress, rigidly, discriminations and perceived threats. Furthermore, a weak but statistically significant negative correlation was observed between harmful leadership and nurses’ overall effectiveness. This finding indicates that nurses, by employing professionalism and critical thinking, were able to buffer the adverse effects of toxic leadership on their performance. The study also identified a significant negative effect of abusive supervision on the nurses’ active engagement. This association may be attributed to characteristic behaviors of oppressive supervisors, including public belittlement of staff and persistent reminders of past errors and failures. Such actions serve as workplace stressors, eroding employees’ psychological resources and well-being. Consequently, staff exposed to corrosive supervision is more inclined to disengage and exhibit silence in the workplace, thereby reducing their levels of professional and organizational commitment.

Organizational silence among nurses and its repercussions, is a major problem addressed by Berma et al. [60] in their research, which took place in Egypt and had a total of 235 participants. This study underscored that workplace toxicity may originate from either managerial figures or colleagues, highlighting multiple potential sources of a detrimental work environment. One quarter of the nurses exhibited high levels of organizational silence as a coping mechanism. Organizational silence—a phenomenon characterised by the reluctance of the employees to share their thoughts and ideas about organizational issues- may stem from various concerns, including fear of losing professional respect, harming relationships with senior management, an inability to openly discuss work-related issues, the presence of a bureaucratic and non-transparent hospital system, fear of punitive responses, self-neglect, inadequate organizational support, and former experiences of abusive supervision. Additionally, although the majority of the nurses were highly committed and passionate to thrive, workplace toxicity fosters increased organizational silence, hindering professional growth and productivity, and ultimately contributing to higher turnover intentions.

Budak & Erdal [61] examined the role of burnout syndrome as a mediator in the link between malevolent leadership and job satisfaction in healthcare environments. Utilizing a sample of 412 participants employed in public hospitals in Turkey, the results revealed that ego-driven and self-serving leadership appears to exert a significantly negative impact on job satisfaction related to managerial roles. Furthermore, it was found to be associated with aspects of burnout encompassing exhaustion, depersonalization, and decreased sense of personal achievement. Burnout resulting from problem-solving demands and efforts to contribute meaningfully also appeared to be influenced by counterproductive leadership practices.

Mrayyan [62] posited in her research in Jordan, the evident ramifications of destructive nursing leadership in workforce preservation. Involving 384 respondents, this study investigated the presence of toxic leadership among nursing leaders in Jordan and its relationship with nurses’ workplace satisfaction, job engagement, and turnover intention. Destructive leadership was identified as a critical determinant of reduced workplace satisfaction and diminished job engagement among nurses, primarily through the cultivation of a dysfunctional and unbalanced work environment. Such conditions contribute to both emotional disconnection and physical disengagement, driven by the sustained intensity and demands of the nursing role. Despite these adverse effects, many nurses demonstrated reluctance to leave their positions. This behavior may be attributed to a cost–benefit analysis shaped by financial constraints and familial obligations, which make employment mobility impractical. While a subset of nurses continued to exhibit professional pride and emotional investment in their work, overall job engagement appeared to be neither spontaneous nor sustainable under persistent tyrannical leadership. The findings of this study concluded that this dynamic poses significant managerial concerns, as prolonged dissatisfaction may ultimately lead to increased turnover intentions and potential attrition from the nursing profession.

Labrague [63] carried out a research study in Philippines, utilizing a sample of 283 nurses. The study aimed to emphasize the direct and mediated influences of harmful leadership styles on occupational satisfaction and psychological discomfort, with work–family conflict serving as a mediating variable. The findings of the study demonstrated that authoritarian and oppressive leadership behaviors exhibited by nurse managers, exert a substantial detrimental effect on employee contentment and its influence on psychological well-being among nurses in emergency settings A critical insight from the analysis was that work–family conflict has partial mediating effects in the dynamic between malicious leadership and these adverse outcomes. This indicates that toxic leadership not only has a direct detrimental effect on nurses’ well-being, but also indirectly exacerbates dissatisfaction and psychological strain by intensifying conflicts between professional and personal roles.

Farghaly Abdelaliem & Abou Zeid [64] in their study in Egypt evaluated the relationship between corrosive leadership and organizational functionality among nursing professionals in a university-affiliated hospital, while also examining how organizational silence mediates this relationship. The study identified a significant inverse relationship between dictatorial leadership and organizational performance. Furthermore, a pronounced negative correlation was observed between unethical leadership and nurses’ organizational silence, indicating that these leadership styles detrimentally affect both individual job satisfaction and the propensity of nurses to withhold their opinions. Organizational silence was found to act as a mediating factor in the relationship between arbitrary leadership and nurses’ organizational performance. Furthermore, a negative correlation was identified between dictatorial leadership and organizational performance.

## 4. Discussion

The findings from this study indicated that toxic leadership behaviors like self-centeredness, low emotional intelligence, rigidity, and excessive competitiveness play a major role in creating and maintaining a culture of silence within healthcare organizations. Furthermore, leadership practices that instill fear are associated with organizational silence, which, in turn, adversely impacts individual development and overall productivity. These dynamics collectively foster increased turnover intentions among nurses [47,49,60,64]. These findings are consistent with the study by Lukacik and Bourdage [65], which similarly identified a correlation between abusive supervision and heightened levels of self-promotion and intimidation. A growing body of literature across various disciplines further supports the assertion that destructive leadership is significantly linked to reduced employee voice. Under such leadership, employees are more inclined to withhold their opinions or merely reiterate the perspectives favored by their superiors, thereby impeding organizational learning and growth [66,67,68]. Kazmi et al. [69] in their research also found that despotic leadership fosters increased employee withdrawal and silent acquiescence, mediated through leader-member exchange and work–life quality.

Furthermore, the study showed that authoritarian and laissez-faire leadership styles act as toxic traits that contribute to autonomy frustration, burnout, lower work engagement, higher turnover intentions, reduced job satisfaction, and greater work-related stress among nurses [48,51,56,63]. These findings align with the research of Schaubroeck et al. [70], who demonstrated that authoritarian leadership exerts a detrimental impact on employee performance, organizational commitment, and intention to remain with the organization. However, the results partially diverge from those of Chen et al. [71], who found that while authoritarian leadership may impede work performance through the activation of hindrance stressors, it may simultaneously enhance performance by eliciting challenge stressors, contingent upon the leader’s power distance orientation. Additionally, the laissez-faire leadership style has been consistently associated, across multiple studies, with adverse outcomes such as heightened job stress and workload, diminished job satisfaction and engagement, and ultimately, reduced staff retention rates [72,73,74,75].

Concerning the impact of toxic leadership on staff retention, research evidence indicates that nursing personnel exposed to such behaviors demonstrate higher absenteeism, elevated stress levels, and stronger intentions to leave the organization.

In contrast, transformational and transactional leadership were associated with higher job satisfaction and lower staff turnover [52,57]. A substantial body of research supports these findings, highlighting that both transformational and transactional leadership styles have been frequently associated with higher job satisfaction and lower rates of employee turnover among nursing professionals [76,77,78,79,80,81].

Moreover, toxic leadership behaviors are markedly associated with lower organizational and professional commitment. This relationship is manifested through poor employee performance, reduced psychological empowerment, and heightened job dissatisfaction, which collectively contribute to both organizational and professional withdrawal [50,54,59]. These findings are consistent with the study by Alsadaan et al. [12], which identified a link between toxic leadership and reduced organizational commitment among nurses. Similarly, Mahgob et al. [82] demonstrated that toxic leadership adversely affects staff nurses’ commitment to their professional roles.

Relevant research indicated a strong relationship between toxic leadership and burnout syndrome—comprising its fundamental elements of psychological fatigue, detachment, and reduced feelings of personal efficacy—and highlighted its detrimental effect on nursing staff retention. These effects ultimately contribute to employee withdrawal, primarily driven by diminished job satisfaction, even though some may be more resilient due to emotional investment and professionalism [61,62]. These results are additionally corroborated by Palvimo et al. [83], who reported a positive association concerning destructive leadership, workplace demands, and burnout in nursing personnel. Similarly, Nunes and Palma-Moreira [84] found that toxic leadership contributes to increased burnout syndrome and turnover intentions. Their study also revealed that disengagement partially mediates this relationship.

Another key finding of the present study is that toxic leadership has adverse effects not only on nurses but also on healthcare organizations, particularly with regard to staff retention. Specifically, it contributes to diminished employee performance, reduced job satisfaction, and weakened extrinsic motivation, all of which adversely impact the quality of organizational services and, ultimately, the institution’s public image [55]. This finding is further supported by the work of Solehudin and Syabanasyah [85], who demonstrated that toxic leadership exerts a negative influence on nurses’ motivation, occupational contentment, output, and staff turnover intentions, ultimately contributing to higher rates of employee attrition. Similarly, Labrague [11] found that toxic nursing leadership is associated with heightened occurrences of adverse events and a decrease in care effectiveness within medical units. Collectively, these studies corroborate the initial conclusion of our study, regarding the detrimental impact of toxic leadership in healthcare organizations.

The study also revealed that counterproductive work behaviors among nurses may serve as a response mechanism to toxic leadership traits, perceived injustice, and experiences of discrimination within the workplace [58]. This finding is further substantiated by multiple studies which have shown that behaviors such as abusive supervision, perceived unfair treatment, and negative management styles directly contribute to an increase in counterproductive work behaviors among nurses. These behaviors are often associated with a rise in adverse events, including patient complaints, medication errors, and healthcare-associated infections. Additionally, affected nurses tend to develop organizational cynicism and harbor negative attitudes towards their work environment [11,86,87,88,89].

**Theoretical and practical implications**

On the theoretical side, the findings of this study provide further confirmation of the Destructive Leadership Model, particularly regarding its defining characteristics and outcomes for both nursing staff and healthcare organizations [90]. Consistent with the model’s description of destructive leadership as systematic and repeated behavior that undermines organizational goals and staff well-being, our review identified multiple examples of toxic leadership in nursing [48,49,50,51,58,59]. For instance, the model emphasizes abuse of power, bullying, intimidation, and humiliation; our findings reveal that nurses experiencing these behaviors report decreased psychological safety, fear of reprisal, and reluctance to voice concerns [47,48,50,53,56,57,58,59,60,63]. Similarly, belittling, humiliation, intimidation and favoritism, also highlighted in the model, were found in our review to erode trust, damage team cohesion, and foster perceptions of inequity and discrimination that drive turnover, lower job satisfaction and commitment [47,49,50,52,54,55,57,58,59,60,61,62,63,64]. In line with the model’s claim that destructive leaders sabotage organizational goals for personal gain and misuse resources, evidence from our review shows that toxic nursing leaders prioritize personal authority and control over patient care quality, thereby compromising organizational effectiveness [48,49,50,51,58,59]. Finally, our findings mirror the model’s predicted outcomes: toxic leadership in nursing was consistently associated with reduced job satisfaction and commitment, heightened stress and burnout, poorer health outcomes among staff and lower productivity [48,49,50,52,53,54,55,56,57,59,60,61,62,63,64]. Collectively, these parallels confirm and extend the Destructive Leadership Model by demonstrating its applicability to the healthcare sector and highlighting the particularly detrimental impact of toxic leadership on nurse retention.

These findings also align with and extend the Job Demands–Resources (JD–R) model, which emphasizes the balance between workplace demands and resources as a key predictor of employee well-being and performance [91]. Toxic leaders intensify psychosocial job demands, including emotional strain, fear, and role conflict, while simultaneously undermining critical job resources such as empowerment, recognition, and support [47,49,50,54,58,59,60]. Within the JD–R framework, this imbalance accelerates burnout and disengagement—outcomes that our review identifies as major contributors to nurse turnover [48,49,50,51,52,56,59,61]. In combination, the Destructive Leadership Model and the JD–R model offer complementary perspectives: the former delineates destructive leadership behaviors and their toxic consequences, while the latter elucidates the mechanisms by which elevated demands and depleted resources translate into burnout and attrition in nursing.

The practical implications of these findings underscore the need for proactive, multi-level interventions to mitigate destructive leadership in healthcare. Mandatory ethical and transformational leadership development programs for middle managers and executives can cultivate constructive behaviors and reinforce a positive organizational culture. Standardized protocols for the early detection of toxic behaviors—incorporating workplace climate indicators, 360° evaluations, and exit interviews—can facilitate timely intervention. Confidential reporting systems are essential to allow staff to disclose abusive behaviors safely, while comprehensive support mechanisms—including psychological counseling, mediation, and, when necessary, temporary reassignment—can protect and assist affected personnel. Finally, integrating leadership metrics into internal quality audits would ensure that leadership practices and personnel management are assessed with the same rigor as clinical outcomes, promoting accountability and sustainable organizational improvement.

## 5. Limitations and Strengths

This scoping review offers additional evidence regarding the relationship between toxic leadership and nursing staff retention, with a key strength being its synthesis of findings from multiple countries, providing a comprehensive and globally informed perspective. Nonetheless, despite the rigorous search and selection strategy employed, some relevant studies may have been excluded, particularly those published in languages other than English, introducing potential publication and language biases. A significant limitation is the regional focus of the studies analyzed, which were predominantly drawn from Asia and Africa, with limited representation from the United States and Europe. Additionally, cultural factors influencing leadership dynamics may shape differing responses to toxic leadership behaviors, potentially affecting the generalizability of the findings. Other limitations include the possible underrepresentation of qualitative studies, which may restrict insight into nurses’ lived experiences, and methodological heterogeneity across the included studies, such as differences in design, measurement tools, and sample characteristics, which may influence the interpretation of results. These limitations highlight the need for future research that incorporates more diverse geographic and cultural contexts, qualitative approaches, and standardized methodologies to strengthen the evidence base and enhance understanding of toxic leadership in healthcare. Longitudinal studies are also suggested to evaluate the impact of interventions designed to mitigate toxic leadership and their effects on nurses’ job satisfaction, retention, and overall well-being over time. Additionally, multicenter comparative studies involving hospitals with diverse organizational cultures would provide insights into how contextual factors influence the prevalence and consequences of toxic leadership

## 6. Conclusions

Exploring toxic leadership in nursing is critical to enhancing nurse job satisfaction, reducing the incidence of adverse patient outcomes, and improving the overall quality of healthcare delivery. By identifying and addressing toxic behaviors, healthcare organizations can create a more supportive and effective work environment for nurses, increase staff retainment and decrease their intention to leave the profession, ultimately benefiting patient care. By identifying specific negative leadership patterns and their consequences on nursing staff retention, this paper provides evidence recommendations for healthcare administrators aiming to foster healthier, more resilient and sustainable workplace environments.

## Figures and Tables

**Table 1:** Search Strategy: Key Terms and Boolean Operators.

Term	Boolean Operator	Term	Boolean Operator	Term
Toxic ^1^ Leadership	AND	Healthcare Organizations		
Toxic Leadership	AND	Nursing Staff		
Toxic Leadership	AND	Healthcare Organizations	AND	Nursing Staff
Toxic Leadership	OR	Nursing Staff	AND	Healthcare Organizations
Healthcare Organizations	OR	Nursing Staff	AND	Toxic Leadership

^1^ The primary search term ‘toxic leadership’ was systematically expanded to include conceptually related terms such as ‘abusive leadership,’ ‘destructive leadership,’ and ‘oppressive leadership’ to ensure comprehensive coverage of the literature.

**Table 2:** Summary of Included Studies’ Characteristics.

Characteristics	Studies *n* (%)
**Area of Studies**	
Europe	2 (11.1)
Asia	8 (44.4)
USA	1 (5.6)
Africa	7 (38.9)
**Year of publication**	
2019	3 (16.7)
2020	2 (11.1)
2021	2 (11.1)
2022	5 (27.8)
2023	3 (16.7)
2024	2 (11.1)
2025	1 (5.6)
**Type of studies**	
quantitative study	10 (55.6)
qualitative study	2 (11.1)
mixed methods	6 (33.3)
**Categorization of studies**	
key dimensions shaping perceptions of toxic leadership	5 (27.8)
impact of toxic leadership on nursing staff retention	13 (72.2)

## Data Availability

No new data were created or analyzed in this study. Data sharing is not applicable to this article.

## Appendix A

**Table A1:** Research studies by Author(s), Country, Aim, Data Sample, Study Design, Data Collection and Data Analysis Method and Finding(s).

A/A	Author(s), Year	Country	Aim	Data Sample	Study Design	Data Collection Method	Data Analysis Method	Finding(s)
1.	Labrague et al., 2020 [52]	Philippines	This study examined the influence of toxic and transformational leadership practices on nurses’ job satisfaction, psychological distress, absenteeism, and intent to leave the organization or the nursing profession.	770	Cross-sectional study	Toxic Leadership Behaviors of Nurse Managers Scale, Global Transformational Leadership, Job Satisfaction Index, Perceived Stress Scale, Two single-item measures developed by O’Driscoll and Beehr, Absenteeism was assessed using a researcher-designed single item question	SPSS Version 22.0	Toxic leadership increased distress and absenteeism; transformational leadership improved job satisfaction.
2.	Hossny et al., 2023 [51]	Egypt	This study was designed to assess nurses’ perception of the effects of organizational climate and toxic leadership behaviors on their intention to stay and the differences in these domains between the two hospitals studied	250	descriptive comparative study	the organizational climate questionnaire (42 items categorized into nine domains), the toxic leadership scale (30 items categorized into five domains), and the Chinese version of the intent-to-stay scale.	IBM SPSS Statistics Version 22.0, Microsoft Excel, GraphPad Prism 5	Positive organizational climate and supportive systems increased nurses’ intention to stay.
3.	Ofei, 2022 [53]	Ghana	This study aimed at assessing the nature and effect of toxic leadership of nurse managers on the perceived job satisfaction and productivity of the nursing workforce.	943	Cross-sectional descriptive study	Toxic Leadership Behaviors of Nurse Managers’ Scale, Perceived Productivity Questionnaire, and the Minnesota Satisfaction Questionnaire (MSQ-short version)	SPSS software version 26	Toxic leadership reduced nurses’ job satisfaction and productivity.
4.	Ramdan and Eid., 2020 [54]	Egypt	This study envisioned to compare toxic leadership among intensive care nursing staff at Tanta University Hospital and El Menshawy hospital and assess its relation to their conflict management style used and organizational commitment at the two hospitals.	544	Descriptive, comparative, cross-sectional study	Toxic Leadership Assessment Scale, Conflict Management Styles, Assessment Scale, Organizational Commitment Assessment Scale	IBM SPSS software package version 20.0.	Toxic leadership reduced organizational commitment and influenced conflict management styles.
5.	Xueqin Guo et al., 2022 [47]	China	The aim of this study is to explore the perceptions of Chinese registered nurses on toxic leadership behaviors of nurse managers and to determine its type, cause and response measures	12	Phenomenological qualitative study	semi-structured in-depth interviews	Colaizzi seven-step analysis method	Nurses working with a transformational leader report higher job contentment and lower intent to leave the nursing profession. Nurses who work for a manager with toxic leadership behaviors demonstrated lower job contentment, higher stress levels, frequent absenteeism and higher intent to leave the nursing profession.
6.	Siyal et al., 2021 [55]	China	The aim of this study is to develop and empirically test a mediation model to examine the indirect impact of abusive supervision on employee performance.	430	Empirical, experimental study	The 10-item scale version of the 15-item scale by Aryee et al., [92] developed by Tepper [93], 4-item scale validated by Amabile et al. [94], general satisfaction 5-item measure by Hackman and Oldham [95], the 4-item scale to measure employee performance developed by Liden et al. [96].	Model development and empirical testing	Abusive supervision reduced employee performance.
7.	Durrah et al., 2024 [48]	France	The current study aims to examine how toxic management styles can lead to both psychological and physical withdrawal of employees in the healthcare sector	413	Quantitative study	Self-developed questionnaire	SmartPLS 3.3.9	Toxic leadership increased both psychological and physical withdrawal behaviors
8.	Trépanier et al., 2019 [56]	Canada	The aim of this paper is to investigate the psychological and motivational processes involved in the relationship between two forms of destructive leadership (tyrannical and laissez-faire) and employee health (burnout, affective commitment and job performance)	399	Cross-sectional study	The Destructive Leadership Scale, The French version of the Psychological Need Thwarting Scale, The Multidimensional Work Motivation Scale, The Maslach Burnout Inventory General Survey, The occupational commitment questionnaire A self-reported scale consisting of 4 items adapted from the in-role performance subscale of the organizational citizenship behavior scale	Structural equation modeling analysis.	Tyrannical leadership frustrates autonomy, competence, and relatedness, leading to burnout and lower performance.
9.	Erschens et al., 2022 [57]	Germany	The aim of this study is to investigate the association of general well-being and different leadership styles among employees in a German tertiary hospital.	1137	Cross-sectional study	Module A and D of the standardized Questionnaire on Integrative Leadership, the five-item World Health Organization well-being index	IBM SPSS version 25	Transformational and transactional leadership styles are associated with higher well-being scores among hospital employees, while laissez-faire and destructive leadership styles are associated with lower scores across all professional groups.
10.	Low et al., 2019 [58]	Malaysia	The aims of this research are to address the two fundamental research questions: (1) What are the antecedents that lead to counterproductive work behavior (CWB) of nurses in public hospitals? (2) How effective are the moderating roles of power distance orientation (a cultural factor) and locus of control (an individual factor) in impacting CWB?	337	Quantitative study	Tepper’s [93] 15-item abusive supervision Measure, Colquitt’s [97] 20-item scale 24-item measure adapted from Mitchell and Ambrose [98] and Bennett and Robinson‘s [99], six-item scale developed by Dorfman and Howell, [100] and Farh, Hackett and Liang’s [101] 16-item Work Locus of Control Scale	structural equation modeling	Abusive supervision leads to counterproductive work behavior in nurses
11.	Shipl et al., 2022 [59]	Egypt	This study aimed to investigate the relationship between toxic leadership and nurse followership effectiveness	343	Cross-sectional study	The Toxic Leadership Scale and the Followership Styles Questionnaire	IBM SPSS, version 25	Toxic leadership negatively correlated with nurse followership effectiveness
12.	Berma et al., 2021 [60]	Egypt	This study aimed to investigate the relationship between workplace toxicity, organizational silence and thriving among nurses.	235	descriptive correlational research	Toxic Workplace Environment Questionnaire, Organizational Silence Scale, Thriving at Work Scale	SPSS version 22.0	Workplace toxicity leads to increased organizational silence, reducing thriving among nurses and potentially leading to staff resignation
13.	Bakkal et al., 2019 [49]	Turkey	The aim of this study is to investigate the effects of the toxic leadership of healthcare employees on the turnover intention and the mediating effects of job satisfaction	658	cross-sectional descriptive study	The Toxic Leadership Scale, the Minnesota Job Satisfaction Questionnaire, a turnover intention scale by Rosin & Korabik [102]	Confirmatory Factor Analysis, Structural Equation Model SPSS 24.0 and AMOS 24.0	Toxic leadership negatively impacts job satisfaction, which in turn increases turnover intention among healthcare personnel
14.	Budak & Erdal, 2022 [61]	Turkey	The aim of this study is to investigate the mediating effect of burnout syndrome on toxic leadership and job satisfaction	412	cross-sectional study	Toxic Leadership Scale, Burnout Scale, Job Satisfaction Scale	Structural Model Analysis	Toxic leadership negatively affects job satisfaction and increases burnout syndrome
15.	Mrayyan, 2025 [62]	Jordan	The aim of this research is to investigate nursing leaders’ toxic leadership, nurses’ workplace satisfaction, job engagement, and turnover intention in Jordan and whether toxic leadership and sample characteristics predict nurses’ work- place satisfaction, job engagement, and turnover intention.	384	cross-sectional study	Toxic Leadership Scale, Nursing Workplace Satisfaction Scale, Job Engagement Scale, Turnover Intention Scale	Online survey, SPSS program version 25	Toxic leadership results in low job satisfaction, stress and emotional exhaustion, and, in turn, decreased quality of nursing care
16.	Labrague, 2024 [63]	Philippines	The aim of this study is to examine the mediating effects of work-family conflict on the relationship between toxic leadership behaviors of nurse managers and psychological distress and work satisfaction among emergency nurses.	283	cross-sectional study	Toxic Leadership Behaviors of Nurse Managers Scale, Work-Family Conflict Scale, Job Stress Scale and the Job Satisfaction Index	Mediation analyses were conducted using the PROCESS Macro with Model 4.	Toxic leadership reduced work satisfaction and increased psychological distress
17.	Farghaly Abdelaliem & Abou Zeid, 2023 [64]	Egypt	The aim of this study is to assess toxic leadership and organizational performance among nurses of a University Hospital, and explore the mediating effect of nurses ‘silence	750	cross-sectional study	The toxic leadership scale, the organizational performance questionnaire	structured equation modeling	Toxic leadership had a significant negative relationship with organizational performance and the nurses’ silence
18.	Ofei et al., 2023 [50]	Ghana	The aim of this study is to investigate the mediating role of job satisfaction on toxic leadership and turnover intentions of nurses	943	cross-sectional study	The Turnover Intention, Minnesota Satisfaction Scale and the Toxic Leadership Behaviors of Nurse Managers’ Scale	SPSS software version 26, descriptive and differential statistics	Job satisfaction acts as a mediating factor for toxic leadership behaviour and nurses’ turnover intentions
